# Cell-based optimization of novel benzamides as potential antimalarial leads

**DOI:** 10.1016/j.bmcl.2009.10.050

**Published:** 2009-12-15

**Authors:** Tao Wu, Advait Nagle, Tomoyo Sakata, Kerstin Henson, Rachel Borboa, Zhong Chen, Kelli Kuhen, David Plouffe, Elizabeth Winzeler, Francisco Adrian, Tove Tuntland, Jonathan Chang, Susan Simerson, Steven Howard, Jared Ek, John Isbell, Xianming Deng, Nathanael S. Gray, David C. Tully, Arnab K. Chatterjee

**Affiliations:** a Genomics Institute of the Novartis Research Foundation, 10675 John J. Hopkins Drive, San Diego, CA 92121, USA; b Harvard Medical School, 250 Longwood Ave., Boston, MA 02115, USA

**Keywords:** Malaria, Kinase, Cellular assay, Cyclic amines, Pharmacokinetics, Resistant strains

## Abstract

Screening our in-house compound collection using a cell based *Plasmodium falciparum* proliferation assay we discovered a known pan-kinase inhibitor scaffold as a hit. Further optimization of this series led us to a novel benzamide scaffold which was devoid of human kinase activity while retaining its antiplasmodial activity. The evolution of this compound series leading to optimized candidates with good cellular potency against multiple strains as well as decent in vivo profile is described in this Letter.

## Graphical abstract

**Figure:**
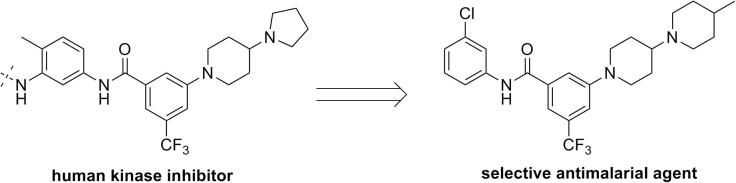

Malaria is an infectious disease that profoundly affects many developing countries. With hundreds of million cases and one million deaths each year, malaria poses a tremendous health and economic burden to the affected regions.^1^ There is still no effective antimalarial vaccine available and we still heavily depend on low molecular weight entities to treat the affected population. Quinine, chloroquine, mefloquine and artemisinin derivatives have play an important role in the treatment of malaria. However, widespread drug resistance has made many of these compounds less effective. Artemisinin is the only anti-malarial for which there are yet no reported cases of clinical resistance. However, parasite tolerance to artemisinin has been observed recently^2^ and it seems likely that resistance will emerge soon. Therefore, it is important to discover new chemotherapies that are effective against the multi-drug resistant parasite strains.^3^ In this Letter, we discuss an effort^4^ to find and optimize novel antimalarial entities using a cell-based screening strategy.

Currently there is a need for novel chemical scaffolds with different mechanisms of action, since most of the current approved antimalarial drugs belong to the aminoquinoline family. In order to find new chemical scaffolds, we initiated a compound screen using our in-house kinase inhibitor collection and subjected them to a cell-based of *Plasmodium falciparum* proliferation assay^5,6^ We envisioned that the hits arising from this screen can be rapidly optimized by leveraging our past experiences with these compounds series in alternative target-classes/indications. Furthermore, we thought that it would be prudent to remove the human kinase activity early on during the compound optimization phase to negate the possibility of toxicity arising from host-related off-target activities (Scheme 1).

Our starting point is compound **1**, which was originally designed as a pan-kinase Bcr-Abl inhibitor^7,8^ Compound **1** shows a moderate EC_50_ of 200 nM against the chloroquine sensitive 3D7 *P. falciparum* parasite strain. By switching the solubility enhancing group, compound **2** exhibits a ∼3-fold improvement in potency. When the 2-methyl group in the left phenyl ring of **2** is replaced with a 3-methoxy group, compound **3** is obtained which is equipotent on malaria parasite. In addition, **3** no longer has any human kinase activities of **2**, as measured in a Ba/F3 transformed cell-line RTK panel^9^ Since preserving the pharmocophore necessary for inhibiting human kinases is not necessary, we speculated that the benzamide portion of the molecule might be responsible for the antimalarial activity of **3** and decided initiate a broad SAR investigation.

Schemes 2–4 describe the synthetic strategies used to study the three key portions of compound **3**. Scheme 2 outlines the synthesis of the amide connectivity of **3**. The synthesis starts from 3-fluoro-5-(trifluoromethyl)benzonitrile. A S_N_Ar reaction followed with a H_2_SO_4_ mediated hydrolysis provides the acid in good yield. The amide bond formation is executed using various amines and HATU as the activating agent. We were satisfied with the straightforward synthesis for these compounds given the need for low cost of goods is one of the essential criteria for the antimalarial target product profile (TPP).^12^

Scheme 3 outlines the synthesis for the reverse amides, sulfonamides and ureas. Curtius rearrangement of the corresponding benzoic acids illustrated in Scheme 2 serves as the key step in the aniline synthetic route.

Scheme 4 outlines the synthesis for determining the SAR on the 3-position on CF_3_ bearing phenyl ring. We started with commercially available 3-bromo-5-(trifluoromethyl)benzoic acid and carried out a palladium catalyzed amination reaction on a variety of substrates to afford the final compounds in moderate to good yields.

Table 1 outline our SAR determination on the amide portion of compound **3**. While linear alkyl (compound **4**) and pyrimidines (compound **5**) are incompatible, a wide variety substituted phenyl groups are well tolerated. The substitution pattern favors meta- and para- mono-substitution, with 3,4-disubstitution (compounds **11)** and 3,5-disubstitution (compound **13**) modestly enhancing potency as well.

Table 2 summarizes how varying the amide linkage affects the potency using compound **8** and **10** as our template since they were potent starting points in our early SAR. The sulfonamide (compound **16**) was not tolerated nor was removal of the carbonyl and replacement with a methylene unit (compound **20**). A free amide NH was essential for activity as the *N*-methyl compound loses potency by 20-fold (compound **17**). Ureas do not seem to offer any advantage over amides, moreover both amide orientations (compound **10** vs **14**) are tolerated and offer advantage in terms of physiochemical properties (e.g., improved solubility). Therefore we decided to explore additional structure–activity relationships with the amide in place.

We next turned our attention to the center benzamide ring to explore the possibility of incorporating heterocycles in that ring. As depicted in Table 3, attempts to make substituent changes (compound **24** and **25**) or incorporate heteroatoms (compound **26**, **27**, **28** and **29**) failed to enhance the potency of the compounds demonstrating tight SAR in that region of the pharmocophore.

One important aspect of antimalarial drug discovery is to identify agents that are active against the drug-resistant parasites. Table 4 outlines our results on chloroquine sensitive 3D7 strain and multidrug resistant W2 strain which is resistant to chloroquine, quinine, pyrimethamine, cycloguanil, and sulfadoxine. While most of these resistance phenotypes are due to point mutations in the drug target, the W2 strain also contains an amplification of the *P. falciparum* multidrug resistance transporter.^10^ Interestingly, while compound **8** show a ∼6-fold shift in potency between 3D7 and W2, replacement of the bicyclic amine part pyrrolidinyl piperidine to bipiperidine (compound **30**, **31**, and **32**) led to much improved potency against W2 strain. Although **34** was the most potent compound, it was deprioritized since the introduction of a stereocenter in the molecule increases the complexity of the synthesis and cost of goods.

Compound **32** was profiled in an extended panel of 15 drug resistant strains (Table 5) and against a 6-cell line toxicity panel. We were delighted to find that all the potencies are within 3-fold of each other. The observed cytotoxicity TC_50_s (293T, Ba/F3, CHO, HEp2, HeLa, Huh7) were greater than 8 μM which translates to a good selectivity index (SI >20).

Some of the more potent compounds against both 3D7 and W2 strains were selected to assess their in vivo pharmacokinetic profiles in mice. Mice were dosed a single dose of 20 mg/kg orally and their exposure levels were monitored over a period of 5 h and results are summarized in Table 6.^11^

Most compound demonstrated good oral exposure, with compound **32** exhibited the highest AUC_(0–5h)_ as well as a high *C*_max_ (∼13-fold above the 3D7 potency). We also performed preliminary solubility and metabolic stability measurements on compound **32**. Compound **32** exhibits moderate solubility (HT-thermodynamic solubility at pH_6.8_ 0.026 mg/mL free base) and modest metabolic stability (extraction ratio in mouse microsomes = 0.383 ± 0.04 and CL_int_ (μL/min/mg) = 21.399) that are consistent with the in vivo pharmacokinetic results.

In conclusion, we have successfully transformed a pan human kinase inhibitor scaffold (compound **2**) into a selective anti-malarial series. SAR studies revealed that the amide linkage is preferred for the antimalarial activity. Switching the bicyclic amine moiety from a 6,5 system to a substituted 6,6 system significantly improves the potency shift across the drug-resistant strains. The selected lead compound **32** exhibited good potency across different strains, favorable physiological properties and good in vivo pharmacokinetic profile. The further studies on this scaffold are warranted and would be reported in due course.

## Figures and Tables

**Scheme 1:**
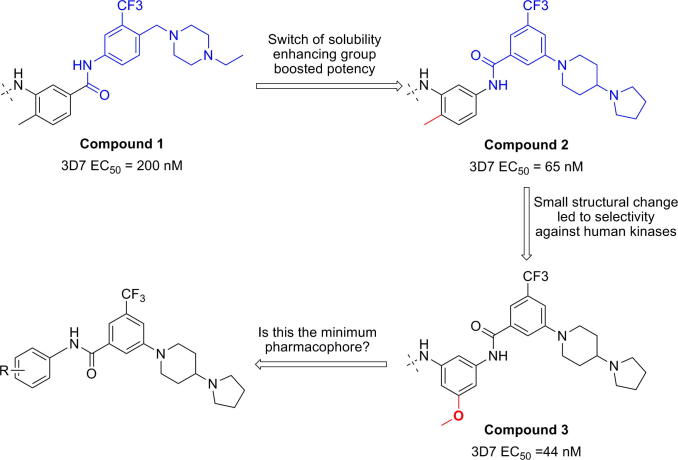
Proprietary kinase scaffolds offered hits: piperidine benzamides.

**Scheme 2:**
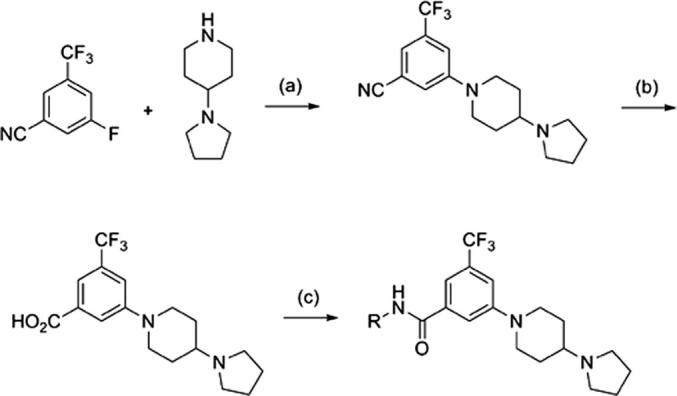
General synthesis scheme of piperidyl benzamides amides. Reagents and conditions: (a) K_2_CO_3_, DMSO, 80 °C; (b) 50% H_2_SO_4_, reflux, 78%, 2 steps; (c) RNH_2_, HATU, DIEA, DMF, 23 °C, 60–70%.

**Scheme 3:**
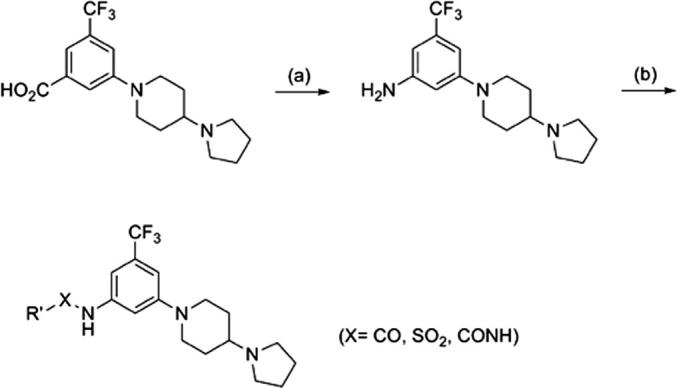
General synthesis scheme of piperidyl benzamides reverse amides, sulfonamides and ureas. Reagents and conditions: (a) (i) DPPA, Et_3_N, *t*-BuOH, reflux; (ii) TFA, DCM, 23 °C, 33%; (b) R′CO_2_H, HATU, DIEA, DMF, 23 °C; or R′SO_2_Cl, DIEA, CH_2_Cl_2;_ or R′NCO, toluene, 80 °C, 40–80%.

**Scheme 4:**
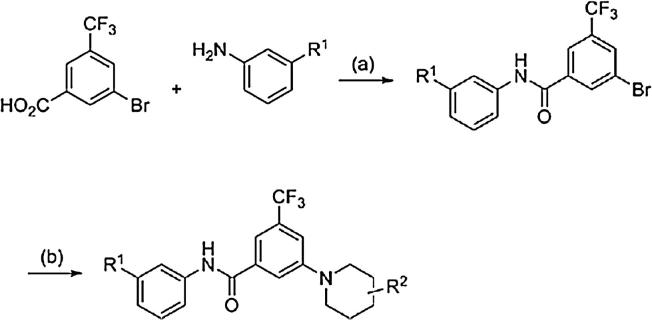
General synthesis scheme of piperidyl benzamides amine SAR. Reagents and conditions: (a) (i) SOCl_2_, CHCl_3_, reflux; (ii) aniline, pyridine, 72–79%; (b) Pd_2_(dba)_3_, BINAP, *t*-BuOK, toluene, 100 °C, 45–84%.

**Table 1:** Piperidyl benzamides SAR: aniline modifications

**Figure:**
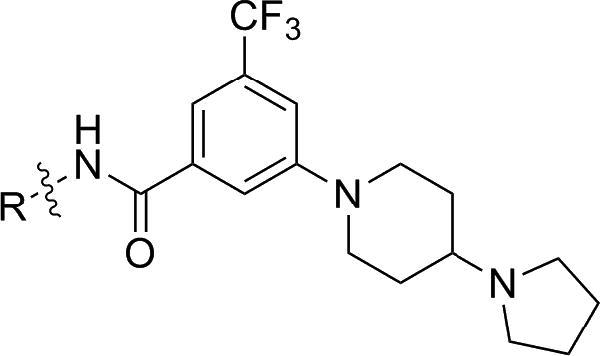

Compds	R	*P. falciparum* 3D7 strain EC_50_, μM^a^
**4**		6.48
**5**		2.16
**6**		1.72
**7**		0.114
**8**		0.134
**9**		0.146
**10**		0.174
**11**		0.054
**12**		0.098
**13**		0.048

a Values are means of two experiments. Each assay plate has mefloquine, sulfadoxine and artimesinin as internal standards. The EC_50_ values for standard compounds match the literature values.

**Table 2:** Piperidyl benzamides SAR: linker modifications

**Figure:**
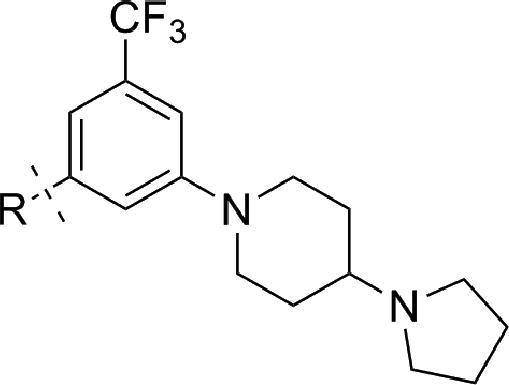

Compds	R	*P. falciparum* 3D7 strain EC_50_, μM^a^
**14**		0.201
**15**		0.402
**16**		8.25
**17**		4.68
**18**		0.452
**19**		0.328
**20**		1.98
**21**		0.638
**22**		0.734
**23**		0.423

a Values are means of two experiments. Each assay plate has mefloquine, sulfadoxine and artimesinin as internal standards. The EC_50_ values for standard compounds match the literature values.

**Table 3:** Piperidyl benzamides SAR: center ring modifications

**Figure:**
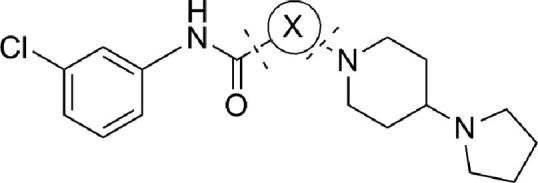

Compds	X	*P. falciparum* 3D7 strain EC_50_, μM^a^
**24**		0.766
**25**		5.88
**26**		0.674
**27**		1.67
**28**		2.98
**29**		2.08

a Values are means of two experiments. Each assay plate has mefloquine, sulfadoxine and artimesinin as internal standards. The EC_50_ values for standard compounds match the literature values.

**Table 4:** Piperidyl benzamides SAR: bicyclic amine modifications

**Figure:**
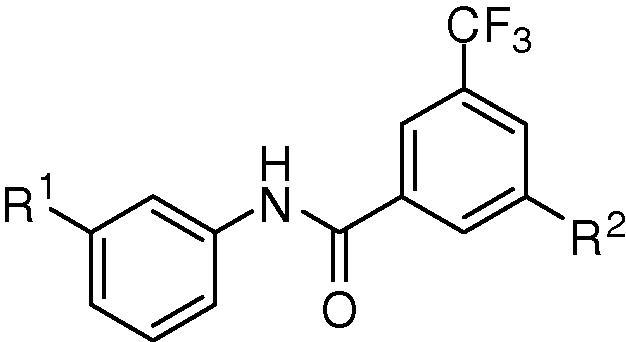

Compds	R^1^	R^2^	*P. falciparum* 3D7 strain EC_50_, μM^a^	*P. falciparum* W2 strain EC_50_, μM^a^
**8**	Cl		0.134	0.888
**30**	Cl		0.082	0.296
**31**	CF_3_		0.149	0.299
**32**	Cl		0.140	0.323
**33**	CF_3_		1.49	1.41
**34**	CF_3_		0.058	0.211
**35**	CF_3_		0.175	0.635

a Values are means of two experiments. Each assay plate has mefloquine, sulfadoxine and artimesinin as internal standards. The EC_50_ values for standard compounds match the literature values.

**Table 5:** Potencies of compound **32** against 15 *P. falciparum* strains

**Figure:**
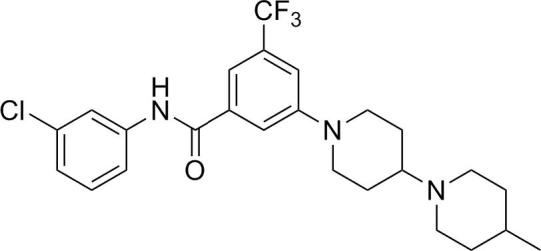

*P. falciparum* strain	EC_50_, μM
3BAG	0.268
7G8	0.309
C188	0.083
D10	0.094
D6	0.081
Dd2	0.141
Camp R	0.138
FCB	0.306
FCR3	0.218
HB3	0.146
K1	0.150
NF54	0.104
3D7	0.140
TM91C235	0.120
W2	0.323

^a^ Values are means of two experiments. Each assay plate has mefloquine, sulfadoxine and artimesinin as internal standards. The EC_50_ values for standard compounds match the literature values.

**Table 6:** Pharmacokinetic profiles of selected compounds

Compds	AUC_(0–5 h)_ (h nM)	*C*_max_ (nM)	*T*_max_ (h)	AUC_(0–5h)_/dose [(min ug/mL)/(mg/kg)]
**8**	3916	1355	1.00	5.31
**13**	4806	1643	5.00	7.01
**14**	4766	1170	3.00	6.94
**30**	4365	1368	1.00	6.10
**32**	7932	2279	5.00	11.42
**35**	1672	672	0.50	2.33

Dose (mg/kg): 20; strain: mice balb/c. Formulation: 2.5 mg/mL in: PEG300 /D5 W, 3:1, solution. Salt form: free base.
